# Congenital Glucose-Galactose Malabsorption: A Case With a Novel SLC5A1 Mutation in a Saudi Infant

**DOI:** 10.7759/cureus.18440

**Published:** 2021-10-02

**Authors:** Loujen O Alamoudi, Albaraa T Alfaraidi, Samiyah S Althagafi, Majid S Al-Thaqafy, Mohammed Hasosah

**Affiliations:** 1 College of Medicine, King Saud bin Abdulaziz University for Health Sciences, King Abdullah International Medical Research Centre, King Abdulaziz Medical City, Ministry of the National Guard - Health Affairs, Jeddah, SAU; 2 Pediatrics, Al Hada Armed Forces Hospital, Taif, SAU; 3 Infection Prevention and Control, Ministry of the National Guard - Health Affairs, Jeddah, SAU; 4 Pediatric Gastroenterology, Ministry of the National Guard - Health Affairs, Jeddah, SAU

**Keywords:** infantile diarrhea, slc5a1, glucose-galactose malabsorption, ggm, autosomal recessive

## Abstract

While only a few hundred cases have been reported in pediatrics, congenital glucose-galactose malabsorption (GGM) is an extremely rare autosomal-recessive metabolic disorder that is characterized by intractable diarrhea and severe dehydration, which can be life-threatening if not treated appropriately. Due to the rarity of the disease, it is challenging to consider GGM as an initial diagnosis for most clinicians. We report the clinical and diagnostic course of a seven-month-old Saudi infant who presented with severe recurrent episodes of watery diarrhea and failure to thrive in early infancy despite standard treatment. Molecular testing identified that our patient had a compound heterozygous variant in *SLC5A1*. Fructose-based formulae have been proven to be effective in treating GGM. This case highlights the importance of early diagnosis and timely management to prevent serious complications of undiagnosed GGM.

## Introduction

Glucose-galactose malabsorption (GGM) (OMIM: 606824) is an extremely rare autosomal-recessive metabolic disorder that is characterized by a defect in transporting glucose and galactose across the intestine border [1]. Individuals with GGM carry two copies of the defective SLC5A1 gene, which is located in chromosome 22q13.1. In healthy infants, the intestinal sodium-dependent glucose cotransporter (SGLT1) drives glucose and water against their concentration gradients into enterocytes via Na+ and electrical gradients [2]. Mutations in SLC5A1 gene lead to a defect in SGLT1 across the intestinal brush border, which results in accumulation of unabsorbed sodium, glucose, and galactose inside the intestinal lumen [3]. GGM is characterized by neonatal onset of severe watery diarrhea that is fatal within few weeks without proper management [2]. Even though there is no cure for GGM, it can be managed by eliminating lactose, glucose, and sucrose from diet. Infants diagnosed with GGM prenatally may thrive on fructose-based formula and subsequently will continue on a fructose-based solid diet [1]. Epidemiological data for GGM are lacking, with only 107 documented cases from 2001 to 2019 worldwide [3], and it is challenging for clinicians to consider GGM as an initial diagnosis. Here we present the clinical and diagnostic course of a Saudi infant with GGM found to have a heterozygous mutation in SLC5A1 gene.

## Case presentation

We present here the case of a seven-month-old boy who is a product of spontaneous vaginal delivery at term; pregnancy, labor, and delivery were uncomplicated. He was born to non-consanguineous parents and has two older healthy siblings with no significant relevant family history. The infant presented to the emergency room multiple times for severe diarrhea episodes that started since the age of four months. He passed watery stools around 10-15 times a day without improvement that was unrelated to acute sickness. The patient was exclusively formula-fed initially but his symptoms significantly increased once solid food was introduced at the age of six months. There was no history of vomiting, poor feeding, or recurrent infections. His development and immunizations were up to his age.

Upon physical examination, he was underweight and looked thin and pale. His weight was below the third percentile with a significantly protruded abdomen, but no hepatomegaly or splenomegaly noted. Physical examination of other systems was unremarkable. He had undergone extensive diagnostic workup including basic routine labs, sweat chloride test, anti-tissue transglutaminase IgA, food and cow milk allergy testing, and upper gastrointestinal endoscopy, which all came normal without abnormalities as shown in Table 1. Neutropenia in our child might be explained by folate and vitamin B12 deficiency (serum levels of these minerals are not shown in table). Various dietary restrictions including a gluten-free diet were tried without improvement.

**Table 1:** Complete Blood Count With Differential Hgb, hemoglobin; RBC, red blood cells

Test Name	Result	Unit	Reference Range
Mean Corpuscular Hgb Concentration	33.4	g/dL	31.9-35.2
Platelet Count	223	10^9^/L	140-450
Mean Platelet Volume	10.7	fL	8.6-12.3
Eosinophils #	0.01	10^9^/L	0.1-1.1
Nucleated RBC %	0.0	%	0-0
Red Cell Count	5.41	10^12^/L	3.7-5.3
Red Cell Distribution Width	13.9	%	11.5-15.3
Lymphocytes #	4.78	10^9^/L	2.5-13.5
Basophils #	0.01	10^9^/L	0-0.1
Hemoglobin	13.7	g/dL	10.5-13.5
Hematocrit	41.0	%	33-39
Mean Corpuscular Volume	75.8	fL	70-86
Mean Corpuscular Hgb	25.3	pg	23-31
White Cell Count	5.82	10^9^/L	5.5-17
Neutrophils #	0.66	10^9^/L	1.5-10
Monocytes #	0.36	10^9^/L	0.2-1.1

Genomic DNA was extracted from peripheral blood and sent for whole-exome sequencing (WES). WES identified the pathogenic heterozygous variant c.765C>G p.(Cys255Trp) in SLC5A1 (OMIM: 182380), which has already been described in the literature with GGM [4]. WES also identified another heterozygous variant c.899G>A p.(Arg300His) in SLC5A1 (OMIM: 182380) reported as likely pathogenic, which has not been described in the literature so far. After a diagnosis of GGM has been confirmed, the infant was started on special formula (Galactomine® - fructose based) along with a suitable dietary plan. The infant is currently thriving well with little-to-no diarrheal episodes.

## Discussion

SLC5A1 gene codes for the SGLT1 that is located on the brush border of the intestinal epithelium [4]. SGLT1 is responsible for the movement of glucose, amino acids, vitamins, and several ions across the intestinal epithelium [5]. GGM occurs secondary to a defect in this intestinal SGLT1; however, fructose and xylose absorptions are not affected. The accumulation of unabsorbed sodium, glucose, and galactose inside the intestinal lumen leads to osmotic diarrhea and severe dehydration. This may be fatal in newborns with GGM if appropriate treatment was not provided promptly. Intolerance to carbohydrates due to genetic causes may have an early or late onset. Carbohydrate intolerances with early onset include GGM, congenital sucrase‐isomaltase deficiency, and congenital lactase deficiency. Intolerance to carbohydrates with late onset includes adult-type lactose intolerance [6].

GGM should be suspected if the following are present: (1) watery diarrhea with onset soon after birth; (2) evidence of carbohydrate malabsorption with positive reducing substance in stool; (3) failure to improve with lactose-free and amino-acid-based formula; (4) improvement of diarrhea only with the elimination of glucose and galactose; (5) exclusion of infections [2,6]. The trials of different milk formulae including lactose-galactose-free formula explain the late presentation of our patient. Furthermore, the population from which the child came is known to have GGM presented at this age [7].

Over 40 variants of SLC5A1 have been associated with GGM [8]. WES of our patient identified a pathogenic heterozygous variant c.765C>G p.(Cys255Trp) in SLC5A1 gene. This variant has been previously reported in the literature to cause GGM [4]. Another heterozygous variant has been identified c.899G>A p. (Arg300His) in SLC5A1 (OMIM: 182380), which has been reported to be likely pathogenic; however, it has not been described in the literature before.

Consanguinity between parents plays a major role in inheriting the disease-causing mutation since GGM is an autosomal-recessive disease. Lee WS reported the first incidence of GGM from Malaysia in 1997 [9]. Data are lacking regarding the incidence or prevalence of this disease. A recent review by Wang et al. reported that out of 107 reviewed cases, 55 cases were from Saudi Arabia and Turkey, accounting for 78.2% of the total number of published cases [3]. Physicians should be vigilant in those countries when an infant presents with intractable, profuse watery diarrhea and dehydration along with failure to thrive that persist regardless of standard therapy. Although infants with GGM present with watery diarrhea after the initiation of milk feeds within a few days after birth, symptoms such as abdominal distention and colic might be present [10]. Moreover, intermittent glycosuria may be observed in GGM patients, a finding that was noted in a Pakistani infant [11].

If left untreated, infants may develop nephrolithiasis and nephrocalcinosis from chronic dehydration, which has been documented in a case series of five Arab children with GGM. As a result of the hypernatremia and dehydration, one of the children developed gangrene in both legs that required bilateral amputation [10]. Renal stones were also observed and reported by Meeuwisse in 1969; however, their cause is still unknown [12]. 

As for the diagnosis, breath hydrogen concentration measurement after glucose consumption can be diagnostic; however, it is unreliable in infants. Total remission of diarrheal symptoms after complete elimination of glucose and galactose from diet can be used for a tentative diagnosis of GGM, which has been adequately shown by a case reported of a Malaysian infant [13]. Hydrogen breath test with glucose or galactose or the oral glucose/galactose tolerance test has been replaced by modern genetic testing. A biopsy of the small intestine with normal histopathology can rule out other causes of malabsorption; however, it is not necessary if the diagnosis can be confirmed with less-invasive tests. Identification of mutations in SLC5A1 gene by WES confirms the diagnosis [14].

The diagnosis of GGM is challenging due to the rarity of the disease. High clinical suspicion is essential and immediate treatment should be provided once a diagnosis of GGM is confirmed. Fructose-based formula has shown to be an effective treatment with dramatic response in infants by leading to cessation of diarrheal episodes and promoting normal neurological development and growth. Parental education regarding GGM dietary management is an important component of the infant's nutrition therapy [15]. Genetic counseling regarding future children may be offered to parents with a family history of newborns affected by GGM especially if consanguinity is present. Prognosis of infants with GGM is better if the diagnosis has been established in early neonatal period since intolerance to glucose and galactose is expected to improve with age [15]. Long-term nutritional management remains a challenging process that requires compliance to special dietary plan and monitoring long-term consequences of following a high-protein and -fat diet.

## Conclusions

The infant has been asymptomatic on fructose-supplemented formula. He has been closely followed up by gastroenterology specialists and a nutritionist with ongoing support and education offered to his parents. Early-onset chronic diarrhea in patients with negative workup for most common causes of chronic diarrhea should alert pediatricians to think about rare congenital causes of chronic diarrhea. Genetic testing is highly recommended and helps to identify those patients earlier, thus preventing serious complications and improving clinical outcomes.
